# Helps in Practice-Building

**Published:** 1904-03-15

**Authors:** A. J. Flanagan

**Affiliations:** Springfield, Mass.


### HELPS IN PRACTICE-BUILDING.

BY A. J. FLANAGAN, D.D.S., SPRINGFIELD, MASS.

Dentistry to-day has decidedly a business as well as a technical side. Professional men are proverbially poor business men. I have frequently thought our colleges should add an extra professorship to be known as that of "Business Methods." The professor should lecture at least once every week, and pay especial attention to those refined minds laboring under the delusion that to exhibit business acumen is to be known as an ethical degenerate. Strange to relate, I have known these "degenerates" in after life to so arise in the average community as to be rated as men of character, reputation, solidity and worth of the foremost rating. It may be well to tell you at this time, that character is what we are, and reputation is what the other fellow says we are. Incidentally the other fellow may be a patient, the dental dealer, your brother practitioner, the cashier at the bank, your landlord, the fellow waiting for the reply to that letter, the insurance man—or even the undertaker.

One day I happened into a large modern mercantile establishment and there noticed many cabinets. I had ransacked many a dental cabinet for pointers, and the habit being still strong, permission was obtained to ransack here. As the result of this visit a business cabinet was soon installed in my office. These cabinets are reasonable in price, can be added to in sections, and can be utilized as individual tastes and ideas may demand. I will enumerate a few sections in my own cabinet: letter file, record file, bills payable file, check file, receipt and note file, periodical file, engagement file, etc. For sections we might mention those of stationery and stock. In cabinets such as these we can put into a compact form many ideas in practice-building of inestimable value.

For some years the essayist has been serving on the staff of a hospital, and we have had several cases there as the result of shock and septicemia following the extraction of teeth. There seems to be a rather common opinion that the extraction of any number of teeth is an easy procedure on the part of the dentists, and the after-treatment and results a something that needs no particular attention on the part of the patient. My candid opinion is, that this opinion on the part of the public never came by intuition but was rather the result of the practitioner's education and action in such matters. If the general surgeon should perform an operation of the simplest kind, and afterward fail to watch, or even prescribe for it, if need be, for a certain length of time, what would the public think of him? To say the least, he would be considered careless. My note-books have a record of several conversations with educated and thoughtful physicians who have frequently been called in to attend patients after the extraction of teeth. Time forbids the relation of these cases and the honest opinions of the cause. My logic would evolve this reasoning: they were not cases of infection from septic hypodermic points or instruments, but simple "simon pure" cases where nothing was prescribed for oral prophylaxis, and no request made on the part of the extractor for a return of the patient for further observation and treatment. A logical reasoning would further evolve the thought that the cases where the physician was called in only the more emphatically proved to the mind of the patient that the dentist was the fellow to employ to "pull," but the physician to treat. An analogous reasoning might bring forth the fact that one was considered a tooth carpenter and the other a tooth doctor. Now all this has a cause and a remedy. It is my practice to impress on my patients the necessity of using a mouth wash, and in certain well-defined cases the need of further treatment either at the office or the home. Time was when I always wrote a prescription for them, but I found many of them neglecting to have the same filled at the chemist's; and so now I invariably put up what is needed in my own office, and the cost is so small that it is hardly worth considering. The mere fact of a proprietary production having a pleasant bouquet and taste does not necessarily mean that it is efficient. My most common wash is of carbolic acid (Scheuring's) two parts, alcohol two parts, glycerine one part. For flavoring you may use oils of peppermint, wintergreen, sassafras or cinnamon. If you wish to impart a pleasing color, add a little red dye. A spoonful of this mixture to one-half pint of water will be found to be about the right proportion. An experience of many years with this preparation has not shown any need of a change. In case you wish an astringent effect, add a small quantity of either fluid red gum or glycerite of tannin. For any case demanding an antiseptic this preparation can be used with results sure to be satisfactory.

If dentistry is regarded as being principally mechanical, we have only ourselves to blame, for the field is wide and limitless for the intelligent and beneficial use of the Pharmacopia. How often we hear patients complain of a feeling of weakness during the long, tedious operations—in fact, do we not frequently have to shorten our operations because of it? Why should not the practitioner use a tonic or stimulant for such cases? If need be, let him direct the use of a tonic three or more days before the appointment. There are many safe and reliable drugs for such purposes. Individually, I prefer strychnia in some form—for I have never known it to fail. We often meet with the opposite of this condition in patients, and there we require a sedative. My most common prescription contains valerian, musk root and asafetida. How many complain of headache following operations, and in the majority of cases a combination of acetanilid, camphor monobromate and citrated caffeine will overcome it. If a dentist has ever had suffering from pericemental trouble, he knows that local instrumentation and application will not always give the desired relief. I know of no one thing in such cases which so helps the practitioner's standing as the intelligent internal administration of drugs to relieve the pain and produce sleep as circumstances demand—the list of reliable preparations is long.

In a general practice we have unfavorable though rarely dangerous symptoms presenting themselves. Here is where cool decision is required—a decision which has in some cases made or unmade a practice. The hospital has taught me to have handy tongue forceps, hemostatics and hypodermic tablets. One corner of my laboratory has a good sized cabinet devoted to drugs and accessories, such as scales, tablet boxes, bottles and labels. It is safe to tell you it has been a good investment for practice-building.

For several years we have had dogmatic teaching, claiming that gold and extention of cavity outline were the two *sine qua nons* of present day practice. These may be scientific truths, but to build a practice on such statements would require wealthy and inanimate patients. Personally, I am always in favor of science, but 'tis frequently found that the other fellow—he at the other end of the instrument—"wouldn't stand for it." Now science is one thing and tact is another. I find a successful practice is not all science of the dogmatics, but frequently fifty percent of the science of human nature. It is still my practice to use plastics, for I have found that in many well-defined cases where gold has been the choice it has been almost impossible to keep my patient quiet long enough to adjust the rubber dam. The vast majority of patients of the present day read and hear so much of what is called "painless dentistry" that a reasonable freedom from pain may be one of the greater essentials in practice-building. Combinations of cement and alloy, tin and gold; cement, alloy and gold; and metal and porcelain inlays are requirements to-day. Many of our patients object strongly to the adjustment of the dam, and to overcome this, it is my habit to use antiseptic napkins held in place by various clamp devices invented by Mr. Ivory, of Philadelphia. After a little practice, it is surprising how little dam you will use when aided by the saliva pump. In the treatment of pulpless teeth you will find that a heater supplied by compressed air in place of a chip-blower will greatly facilitate matters. One of these outfits can be installed easily where a water supply can be had. Other uses of this appliance are in the treatment of sensitive dentine and blowing blood out of the pockets in pyorrhea cases; by attachment to atomizers it can be used in forcing medicaments into these pockets. Besides the napkin-holders mentioned, Mr. Ivory has several varieties of matrices and holders for use with the plastics, and by their use we need have no projections at the gum line and have an adjustable contour at the same time. The dental parlors teach their patients that crown and bridge-work is the part of our calling which demands the most knowledge and skill. It is my frank opinion that if it were not for the small amount of time involved for the greater financial returns, we would not see so much of this jewelry work. If you would have the most intelligent and permenent practice, teach your patients that the saving of the natural teeth represents that part of your profession. Honest, conservative bridge-work, of limited extent, is the teaching of the more experienced men of to-day; a thorough knowledge of mouth antisepsis and the stress or strain on the natural teeth in ordinary mastication will prevent the dentist who desires a permanent practice from going to extremes in bridge-work. That saying of Lincoln's relative to "fooling the people" is most apropos to extreme bridge-work.

For the cheaper forms of crown and bridge-work the Samsioe method will well repay investigation. For temporary work it cannot be surpassed. The setting of crowns and bridges by cement has the one great drawback of not being easily removed. The electric heater and gutta-percha is now coming to the front for use in attaching this work. In case you wish to remove, have a long glass rod of suitable thickness, heat it so that the patient will not see you holding it in the flame, and then hold it upon your crown or bridge until the attachment is made soft enough to loosen. The glass rod holds the heat well, and when heated very hot is not of that red color you would have in using iron or steel; therefore the patient is not liable to fear it. The electric heater is also the best medium for softening gutta-percha for filling purposes.

The smooth-running engine and handpiece (with emphasis on the handpiece), together with sharp burs (again, decided emphasis on sharp), guided by a delicate touch, is still the main reliance for painless excavation of carious matter. There are various medicaments of partial help, but not one that is always to be relied on. An investigation of the handpieces and burs used by the majority of operators convinces me that there lies the greater source of pain. A handpiece needs regular attention to keep it running true, and a bur needs the angle of the cutting blades to be very precise in order to prevent jar and heat of an unnecessary amount. A fair knowledge of the principles of mechanics will demonstrate to you in a few moments the two evils of untrue rotation and the wrong angle of cutting edge in tools. There is a chance in our calling for a very scientific and practical discovery along these lines by some ingenious and educated member.

My notes have many references to the matter of dental knowledge as exhibited by the average patient. The lasting reputation and permanent income from our practice depends to a vast extent on what we impart in the line of dental knowledge and advice to our patients. If there is one word that should be eliminated from our conversation with patients, it is the word "dead." The ordinary idea of that word would signify a something that had passed from existence and could not trouble again. We have had a bad inheritance left us by our older practitioners and it is high time we substitute the word "pulpless." A few old teeth treated in a manner to show the pulp-canals and especially the peridental membranes will give an object lesson far surpassing talk. Old teeth showing the results of calculus and pyorrhea are equally effective. Again, a few specimens of crown and bridge-work and even plate-work serve a very sufficient purpose. The dental parlor charlatans well recognize the value of such things, and it may sound a little "shoppy" to speak of this. My idea is to educate the public to a better and a higher ideal of dentistry, and if the showing and explanation of these kinds of operations while the patient is in your office will accomplish this, we should not hesitate. We always make the sad mistake of thinking our patients can grasp an operation or idea by a few words of explanation. Above all, educate them to the idea that no operation, in some respects, can be considered perfect for all conditions and time. The fakirs are daily doing an irreparable mistake in putting forth guarantees for ten and twenty years.

Last, but not by any means least in the matter of education, be careful of the criticism of your ethical brother's work; remember well that you know not the many conditions which should be taken into account in judging another's work. Remember well that charity does not consist in mere almsgiving, but springs from the soul, a gem of unrivaled value, a something by which the world is made kin. If an educated, refined professional practitioner oversteps the bounds in such matters, what must be the just estimate of the public in events like these?

In plate-work I wish to recommend to you the use of stiff trial plates, such as will not lose their form by ordinary handling and not be softened by the temperature of the mouth.

A thorough study and mastery of many of the principles of articulation as laid down by the late Dr. Bonwill will assist you greatly in enabling your patients to have a comfortable and efficient use of artificial dentures. Time was when I thought the Bonwill articulator was the only true one. We have now a much better and, it seems to me, almost perfect articulator, known as the Kerr. In my prosthetic work I follow Bonwill's principles on the Kerr articulator.

Perhaps you have been wearied enough, and while there are many other ideas to help some member of our calling, I wish to speak of only one—and that is the office environment. First, last, and for all time, special stress should be laid not only on what is known as cleanliness, but—a still better term—asepsis of all surroundings. No matter how lavish the surroundings, if this requisite is lacking, you have naught for your expenditures. I do not underestimate the more modern chairs, cabinets, fountain spittoons, power engines, electric appliances, etc., as assisting in building a practice, but above all these stands the one grand *sine qua non*—asepsis. How many have failed because they had it not—how many succeeded because they possessed it!—*Phila. Dental College Alumni Annual.*

